# Citalopram Ameliorates Impairments in Spatial Memory and Synaptic Plasticity in Female 3xTgAD Mice

**DOI:** 10.1155/2017/1238687

**Published:** 2017-09-18

**Authors:** Zhang Wei, Guo Junhong, Niu Xiaoyuan, Wang Jie, Wang Zhaojun, Wu Meina, Yang Wei, Zhang Jun, Qi Jinshun

**Affiliations:** ^1^Department of Physiology, Shanxi Medical University, Taiyuan, Shanxi 030001, China; ^2^Department of Neurology, First Hospital, Shanxi Medical University, Taiyuan, Shanxi 030001, China

## Abstract

Alzheimer's disease (AD) is the primary cause of dementia. There is no effective treatment. Amyloid-*β* peptide (A*β*) plays an important role in the pathogenesis and thus strategies suppressing A*β* production and accumulation seem promising. Citalopram is an antidepressant drug and can decrease A*β* production and amyloid plaques in transgenic mice of AD and humans. Whether citalopram can ameliorate memory deficit was not known yet. We tested the effects of citalopram on behavioral performance and synaptic plasticity in female 3xTgAD mice, a well-characterized model of AD. Mice were treated with citalopram or water from 5 months of age for 3 months. Citalopram treatment at approximately 10 mg/kg/day significantly improved spatial memory in the Morris water maze (MWM) test, while not affecting anxiety-like and depression-like behavior in 3xTgAD mice. Further, hippocampal long-term potentiation (LTP) impairment in 3xTgAD mice was reversed by citalopram treatment. Citalopram treatment also significantly decreased the levels of insoluble A*β*_40_ in hippocampal and cortical tissues in 3xTgAD mice, accompanied with a reduced amyloid precursor protein (APP). Together, citalopram treatment may be a promising strategy for AD and further clinical trials should be conducted to verify the effect of citalopram on cognition in patients with AD or mild cognitive impairment.

## 1. Introduction

Alzheimer's disease (AD) is a severe neurodegenerative disease characterized by memory deficit and progressive cognitive impairment. It is the most prevalent cause of dementia and affects more than 30 million people all around the world [1]. Despite decades of research efforts, there is no effective treatment for AD. Amyloid-*β* peptide (A*β*) is the major component of amyloid plaques which is the core neuropathological hallmark of AD [2]. Accordingly, A*β* is deeply involved in the pathogenesis and causes synapse failure and neuron loss by increased oxidative damage, impaired energy metabolism, and perturbed cellular calcium homeostasis [3–5]. Therefore, strategies suppressing A*β* production and accumulation seem promising.

A*β* is a 4 kDa peptide produced by endoproteolysis of the amyloid precursor protein (APP) which is achieved by the sequential cleavage of APP by *β*- and *γ*-secretases [4, 6]. First, *β*-secretase cleaves APP to produce a secreted N-terminal soluble extracellular fragment of APP (sAPP*β*) and membrane-bound C-terminal fragments of APP (CTF*β*). Second, *γ*-secretase catalyzes the intramembrane proteolysis of CTF*β* to produce A*β*. APP also can be cleaved by *α*-secretase in a nonamyloidogenic pathway, which prevents the generation of A*β* [4, 6].

Citalopram is an antidepressant drug of the selective serotonin reuptake inhibitor (SSRI) class and used in AD patients for treating depression and related symptoms [7]. Previous studies have shown that citalopram can decrease A*β* production and amyloid plaques in transgenic mice of AD and humans [8, 9]. Furthermore, a history of depression increases the risk for later developing AD [10]. Abnormal serotonergic system may play an important role in the link [11]. In AD patients, serotonin concentrations are reduced in hippocampal cortex and hippocampus [12, 13]. Citalopram can increase hippocampal 5-HT levels in mice [14] and may restore 5-HT levels in AD patients. However, whether citalopram can ameliorate memory deficit was still unknown.

In this study, we tested the effects of citalopram on behavioral performance and synaptic plasticity in female 3xTgAD mice, a model of AD. The model harbors PS1_M146V_, APP_Swe_, and tau_P301L_ transgenes and exhibits age-dependent deficits in spatial memory and synaptic plasticity [15, 16]. Our results showed that chronic administration of citalopram significantly improved spatial memory and synaptic plasticity and decreased insoluble A*β*_40_ levels in female 3xTgAD mice. Together, citalopram treatment may be a promising strategy for AD and further clinical trials should be conducted to verify the effect of citalopram on cognition in patients with AD or mild cognitive impairment.

## 2. Materials and Methods

### 2.1. Animals

The generation of 3xTgAD mice was described previously [15]. The homozygous 3xTgAD mice were purchased from the Jackson Laboratory. Female mice expressed more obvious phenotype in this model of AD [17]. Also in human, female had higher prevalence of AD [18]. To exclude the possibility of citalopram improving cognition by reducing depression, young animals were selected. Thus, experiments were performed using 5-month-old female 3xTgAD mice. The age- and sex- matched wild-type (WT) mice were used as the controls. The mice were maintained under a 12 h/12 h light cycle with free access to food and water and were used in accordance with the guidelines of Shanxi Committee on Ethics of Animal Research.

### 2.2. Drug Treatment

Citalopram (Lundbeck) was given to the mice through the drinking water at a concentration of 0.06 mg/ml (approximately 10 mg/kg/day). A 10 mg/kg/day dose for a mouse is equivalent to 50 mg/day in humans [9, 19], which is within the dose range of citalopram (10–60 mg/day) for treating depression [20]. Mice were treated with citalopram or water from 5 months of age for 3 months and divided into 4 groups: WT + water (*n* = 11), WT + citalopram (*n* = 12), 3xTgAD + water (*n* = 11), and 3xTgAD + citalopram (*n* = 12). The drug treatment did not result in any mortality.

### 2.3. Behavioral Tests

#### 2.3.1. Open Field Test

The open field test (OFT) was used to measure locomotion, exploration, and anxiety-like behavior [14]. The test was assessed at 7 months of age. The apparatus consisted of a square gray box (40 cm × 40 cm × 40 cm), made out of plexiglass. Each mouse was placed in the apparatus for a 10 min period. The total distance travelled in the open field and time spent in inner square (20 cm × 20 cm) were measured. Performance was recorded using a camera above the box and analyzed with a video tracking software (SMART 3.0, Harvard Apparatus, USA). The apparatus was cleaned with 70% ethanol after each trial.

#### 2.3.2. Morris Water Maze Test

The spatial learning and memory of mice were evaluated by the Morris water maze (MWM) test [21, 22]. The test was carried out in a circular tank (diameter 150 cm, height 50 cm) containing tap water one week after the OFT. The temperature of the water was maintained at approximately 22°C. The water in the pool was opaque by nontoxic white paint. Different prominent visual cues were displayed on the inner wall of the tank. The swimming traces of mice were recorded and analyzed by a camera hanging above the tank and a software tracking system (Ethovision 3.0, Noldus Information Technology, Netherlands). For the hidden platform task, a platform (diameter 14 cm, height 29 cm) was set 1 cm below the water surface in the “center” of one quadrant and each animal was trained four times per day for 5 consecutive days (days 1–5). On each training trial, a mouse was placed in the water facing the tank wall with the start quadrants varying pseudorandomly and allowed to swim freely to the platform. When the mouse found the platform, it was allowed to stay on it for 20 s. After then, it was removed to the home cage for 20 s before the next trial. If the mouse did not find the platform within 60 s, it was guided gently to the platform and stayed there for 20 s. The escape latency was used to assess learning ability. On day 6, a probe trial was conducted with no platform in the pool for each animal to evaluate the ability of memory retention by measuring the number of platform crossings. Mice were allowed to swim for 60 s in the pool. To exclude the possibility of genotype or citalopram-induced locomotor deficits, swimming speed was also measured in the probe test. Mice that floated during the MWM test were not included in the final data.

#### 2.3.3. Elevated Plus Maze Test

The elevated plus maze (EPM) test was used to evaluate anxiety-like behavior in mice as described [23]. The test was conducted 20 days after the OFT. The metal apparatus was raised 50 cm above ground and consisted of two opposite open arms (30 × 6 cm) and two opposite closed arms (30 × 6 × 15 cm) which extended from a common central platform (6 × 6 cm). The mice were allowed to move freely in the maze for 5 min. Total distance travelled in the maze and time spent in the open arms were recorded and measured by a camera above the maze and a software tracking system (Ethovision 3.0, Noldus Information Technology, Netherlands). The apparatus was cleaned with 70% ethanol after each trial.

#### 2.3.4. Tail Suspension Test

The tail suspension test (TST) was used to assess depression-like behavior in mice as described [24]. The test was conducted 25 days after the OFT. The mouse was suspended by the tail from a hook in a white-painted box (30 × 20 × 20 cm) for 5 min. Movements were recorded by a camera. Immobility time was measured by a video tracking software (SMART 3.0, Harvard Apparatus, USA). The apparatus was cleaned with 70% ethanol after each trial.

### 2.4. In Vivo Hippocampal Long-Term Potentiation Recording

The effect of citalopram on synaptic plasticity was tested by in vivo hippocampal long-term potentiation (LTP) recording, which has been widely accepted as an electrophysiological neuronal model of memory [25]. The electrophysiological study was conducted 30 days after the OFT. The mice were anesthetized with chloral hydrate (350 mg/kg, i.p.) and placed in a stereotaxic device for acute surgery and LTP recording. A hole with an approximate 2.0 mm diameter was drilled on the skull. A concentric stimulating electrode (FHC, USA) was placed at the Schaffer collateral/commissural pathway (2.0 mm posterior to bregma and 1.5 mm lateral to the midline). A recording electrode was inserted into the CA1 region of the hippocampus (1.5 mm posterior to bregma and 1 mm lateral to the midline) to record field excitatory postsynaptic potentials (fEPSPs) in stratum radiatum. An electronic stimulator (Master-9, AMPI, Israel) and a coupled isolator (ISO-Flex, AMPI, Israel) were used to generate pulse stimulation. The signals from the recording electrode were amplified by a multichannel biological signal acquisition/processing system (Chengdu Instruments Ltd., China). Test stimulation (intensity, 30–50% of maximal EPSPs; frequency, 0.033 Hz) was given for at least 15 min to establish a stable baseline fEPSPs. High-frequency stimulation (HFS) was applied to induce LTP of fEPSP. The HFS consisted of 3 trains of 20 stimuli with an interstimulus interval of 5 ms (200 Hz) and an intertrain interval of 30 s. After the HFS, fEPSPs were recorded at 0.033 Hz for 1 h. The slope of fEPSPs was normalized to basal fEPSPs and averaged. Area under the curve (AUC) that showed time-course response was measured from 0 to 10 min and 21 to 60 min after HFS. Paired-pulse facilitation (PPF) was measured to analyze presynaptic functions before HFS. The interval between 2 stimuli was 50 ms. After LTP recording, the mice were euthanized at once. Brains were rapidly removed and hippocampi were dissected from the brains and immediately frozen and stored at −80°C.

### 2.5. ELISA Analysis of A*β* Levels

Hippocampal and cortical tissues (100 mg/ml wet weight) were homogenized using a dounce homogenizer in a buffer containing 50 mM Tris-HCl, pH 7.4, 150 mM NaCl, 1% Triton X-100, 1% sodium deoxycholate, 0.1% sodium dodecyl sulphate, 1 mM PMSF, a cocktail of protease inhibitors (Boster, China), and a cocktail of phosphatase inhibitors (Boster, China). Samples were allowed to sit on ice for 1.5 h and centrifuged at 13,000 g for 20 min (4°C). Supernatants containing the detergent soluble fraction were collected and stored at −80°C and used for measuring detergent soluble A*β* and western blotting analysis. Pellets (100 mg/mL wet weight) were resuspended by brief sonication in 6 mol/L guanidine-HCl, 50 mmol/L Tris-HCl, pH 7.4 and centrifuged at 13,000 g for 20 min (4°C). Supernatants were collected, stored at −80°C, and used for quantification of insoluble A*β*. Protein concentrations were determined using a BCA kit (Boster, China). The concentrations of A*β*_40_ and A*β*_42_ were measured using a commercial kit, following the manufacturer's protocol (Cloud-Clone, China).

### 2.6. Western Blotting Analysis

The extracts for detergent soluble A*β* ELISA from hippocampal tissues were also used to perform western blotting analysis. Proteins were separated by electrophoresis with the 12% SDS-PAGE and transferred onto PVDF membranes (Boster, China). The blot was probed with A8717 (Sigma, USA) to detect APP and CTF*β* and rabbit anti-*β*-actin (Bioworld Technology, USA) to control for loading differences, followed by peroxidase-conjugated goat anti-rabbit IgG (Boster, China). The protein bands were visualized by ECL detection reagents (Applygen, China). Western blot images were quantitated using ImageJ (National Institutes of Health, USA). The ratios of target proteins over *β*-actin were calculated.

### 2.7. Statistical Analysis

Values were shown as mean ± standard errors (SEM). Differences between 2 means were assessed by Student's *t*-test. Two-way analysis of variance (ANOVA) or three-way repeated ANOVA was used to determine the significant differences between multiple means, followed by Bonferroni's post hoc tests. For all statistical tests, *P* < 0.05 was considered statistically significant. Analysis was performed using the statistical software package SPSS 13.0 or sigmaplot 12.0.

## 3. Results

### 3.1. Citalopram Treatment Ameliorated Behavioral Abnormalities in 3xTgAD Mice

**Figure 1:**
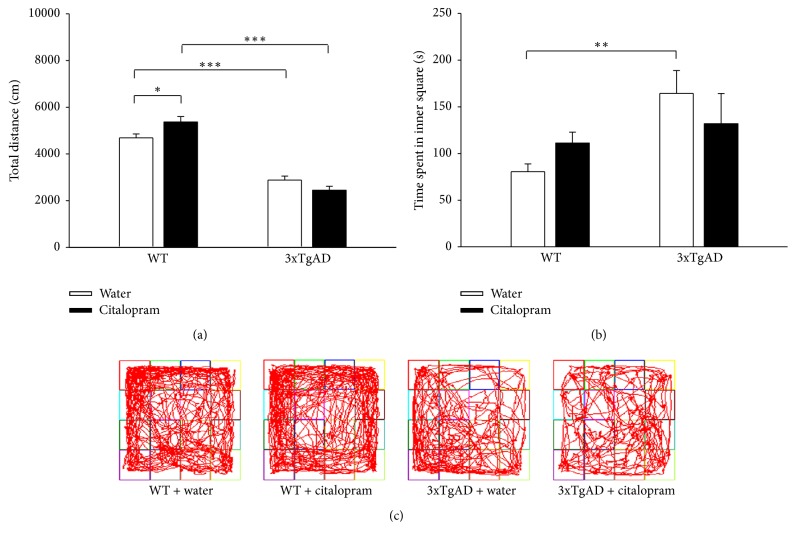
3xTgAD mice showed abnormalities in the open field test at the age of 7-8 months, which were not ameliorated by citalopram. (a) A reduction in locomotor activity was observed in 3xTgAD mice. (b) 3xTgAD mice spent more time in inner square. (c) Representative traces of mice in the open field test. Error bars indicate SEM. ^*∗*^*P* < 0.05, ^*∗∗*^*P* < 0.01, and ^*∗∗∗*^*P* < 0.001.

Locomotion and anxiety-like behavior were first investigated by the OFT. Two-way ANOVA of the OFT showed that total distance of 3xTgAD mice was significantly less than that of WT mice (*F*_(1,41)_ = 150.35, *P* < 0.001 for genotype, *F*_(1,41)_ = 0.383, *P* = 0.540 for citalopram, and *F*_(1,41)_ = 8.242, *P* = 0.006 for the interaction between genotype and citalopram). Post hoc analysis revealed that total distance in 3xTgAD mice was decreased compared to WT mice (*P* < 0.001, *n* = 11; Figure 1(a)). Citalopram treatment significantly increased total distance in WT mice (*P* = 0.017, *n* = 11-12), but not in 3xAD mice (*P* = 0.123, *n* = 11). Two-way ANOVA also indicated that genotype, but not citalopram, had significant main effect on time spent in inner square (*F*_(1,41)_ = 6.069, *P* = 0.018 for genotype, *F*_(1,41)_ = 0.00189, *P* = 0.966 for citalopram, and *F*_(1,41)_ = 2.212, *P* = 0.145 for the interaction between genotype and citalopram). Post hoc analysis indicated that 3xTgAD mice spent more time in inner square than WT mice (*P* = 0.009, *n* = 11; Figure 1(b)). Citalopram treatment had no significant effect on WT mice (*P* = 0.308, *n* = 11-12) or 3xAD mice (*P* = 0.290, *n* = 11). Representative traces of mice in the OFT are shown in Figure 1(c). In OFT, 3xTgAD mice showed reduced motor activity and a lower level of anxiety which were not improved by citalopram treatment.

**Figure 2:**
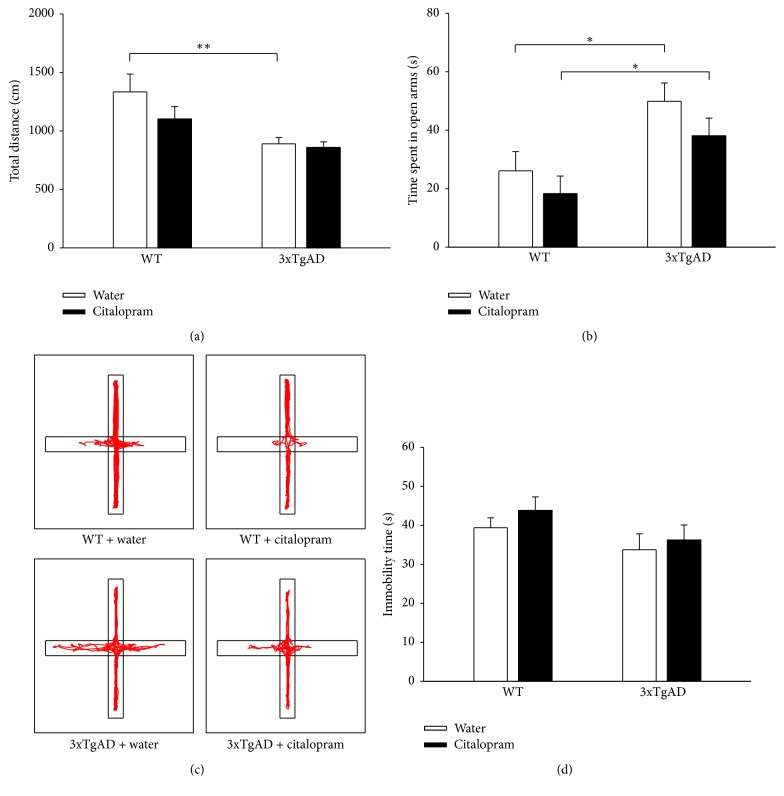
3xTgAD mice showed abnormalities in the elevated plus maze test and did not present depressive-like behavior in the tail suspension test at the age of 7-8 months. (a) In the elevated plus maze test, a reduction in locomotor activity was observed in 3xTgAD mice, which was not improved by citalopram. (b) In the elevated plus maze test, 3xTgAD mice spent more time in the open arms and citalopram did not take effect. (c) Representative traces of mice in the elevated plus maze test. (d) In the tail suspension test, 3xTgAD mice did not exhibit depressive-like behavior. Error bars indicate SEM. ^*∗*^*P* < 0.05, ^*∗∗*^*P* < 0.01.

**Figure 3:**
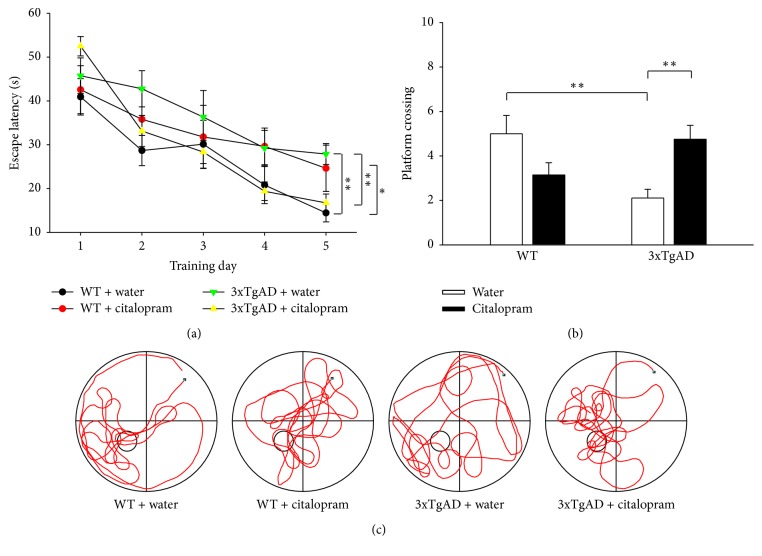
Citalopram treatment ameliorated the impairments in spatial learning and memory of 3xTgAD mice in Morris water maze test. (a) Plots showing the changes in the escape latencies of mice in different groups over the 5-day training period. The escape latency of 3xTgAD mice was longer than that of WT mice on the fifth day, and citalopram reversed it. (b) The number of platform crossings was decreased in 3xTgAD mice, which was reversed by citalopram. (c) Representative swimming traces of mice in the probe test. Error bars indicate SEM. ^*∗*^*P* < 0.05, ^*∗∗*^*P* < 0.01.

Locomotion and anxiety-like behavior were also assessed with the EPM test. In the two-way ANOVA, 3xTgAD mice showed decreased total distance compared to WT mice (*F*_(1,42)_ = 12.247, *P* = 0.001 for genotype, *F*_(1,42)_ = 1.752, *P* = 0.193 for citalopram, and *F*_(1,42)_ = 1.025, *P* = 0.317 for the interaction between genotype and citalopram). Post hoc analysis revealed that total distance in 3xTgAD mice was less than that in WT mice (*P* = 0.003, *n* = 11; Figure 2(a)), and citalopram treatment did not affect total distance in WT mice (*P* = 0.106, *n* = 11-12; Figure 2(a)) or 3xAD mice (*P* = 0.827, *n* = 11-12). Two-way ANOVA of time spent in open arms revealed that genotype, but not citalopram, had significant main effect (*F*_(1,41)_ = 12.258, *P* = 0.001 for genotype, *F*_(1,41)_ = 2.464, *P* = 0.124 for citalopram, and *F*_(1,41)_ = 0.101, *P* = 0.752 for the interaction between genotype and citalopram). Post hoc analysis showed that time spent in open arms was significantly increased in 3xTgAD mice when compared to WT mice (*P* = 0.012, *n* = 10-11; Figure 2(b)), and citalopram treatment did not affect the time spent in open arms in WT mice (*P* = 0.387, *n* = 10–12) or 3xAD mice (*P* = 0.184, *n* = 11-12). Representative traces of mice in the EPM test are shown in Figure 2(c). In EPM test, 3xTgAD mice also showed reduced motor activity and a lower level of anxiety, and citalopram treatment did not affect the behavioral performance.

Depression-like behavior was investigated by the TST. Two-way ANOVA showed that genotype and citalopram treatment had no significant main effect on the immobility time (*F*_(1,42)_ = 3.463, *P* = 0.07 for genotype, *F*_(1,42)_ = 0.959, *P* = 0.333 for citalopram, and *F*_(1,42)_ = 0.0719, *P* = 0.790 for the interaction between genotype and citalopram). Post hoc analysis indicated that the difference of immobility time was not significant between 3xTgAD and WT mice (*P* = 0.276, *n* = 11; Figure 2(d)), and citalopram treatment had no effect on the immobility time in WT mice (*P* = 0.383, *n* = 11-12) or 3xAD mice (*P* = 0.618, *n* = 11-12). Thus, 3xTgAD mice did not present depressive behavior at this age.

The spatial learning and memory of mice were assessed with the MWM test. For the hidden platform task used for evaluating spatial learning ability, three-way repeated ANOVA showed that the escape latency of all mice decreased over the 5-day training period (*F*_(4,128)_ = 20.898, *P* < 0.001; Figure 3(a)). Genotype had significant main effect on the escape latency and a significant interaction between genotype and citalopram existed (*F*_(1,32)_ = 4.217, *P* = 0.048 for genotype, *F*_(1,32)_ = 0.024, *P* = 0.878 for citalopram, and *F*_(1,32)_ = 14.916, *P* = 0.001 for the interaction between genotype and citalopram). Post hoc analysis revealed that 3xTgAD mice spent longer time to find the hidden platform than WT mice over the course of the 5 days (*P* < 0.05, *n* = 8-9). Citalopram treatment resulted in a significantly greater decrease in escape latency in 3xTgAD mice (*P* < 0.05, *n* = 9–12). The results suggested that 3xTgAD mice exhibited impaired spatial learning ability which was reversed by citalopram treatment.

For the probe test used to assess spatial memory, two-way ANOVA showed that there was a significant interaction between genotype and citalopram on the number of platform crossings (*F*_(1,32)_ = 0.998, *P* = 0.325 for genotype, *F*_(1,32)_ = 0.371, *P* = 0.547 for citalopram, and *F*_(1,32)_ = 12.274, *P* = 0.001 for the interaction between genotype and citalopram). Post hoc analysis revealed that the number of platform crossings of 3xTgAD mice was significantly fewer than that of WT mice (*P* = 0.004, *n* = 8-9; Figure 3(b)), and citalopram reversed the effect (*P* = 0.003, *n* = 9–12). During the probe test, swimming speed was comparable between 3xTgAD and WT mice (*P* = 0.566, *n* = 8-9), and citalopram treatment did not affect swimming speed in 3xTgAD (*P* = 0.153, *n* = 9–12) or WT mice (*P* = 0.064, *n* = 7-8). The results indicated that the 3xTgAD mice had deficits in spatial memory which was ameliorated by citalopram treatment. Representative swimming traces of mice in the probe test are shown in Figure 3(c).

### 3.2. Citalopram Treatment Rescued Hippocampal LTP of 3xTgAD Mice

**Figure 4:**
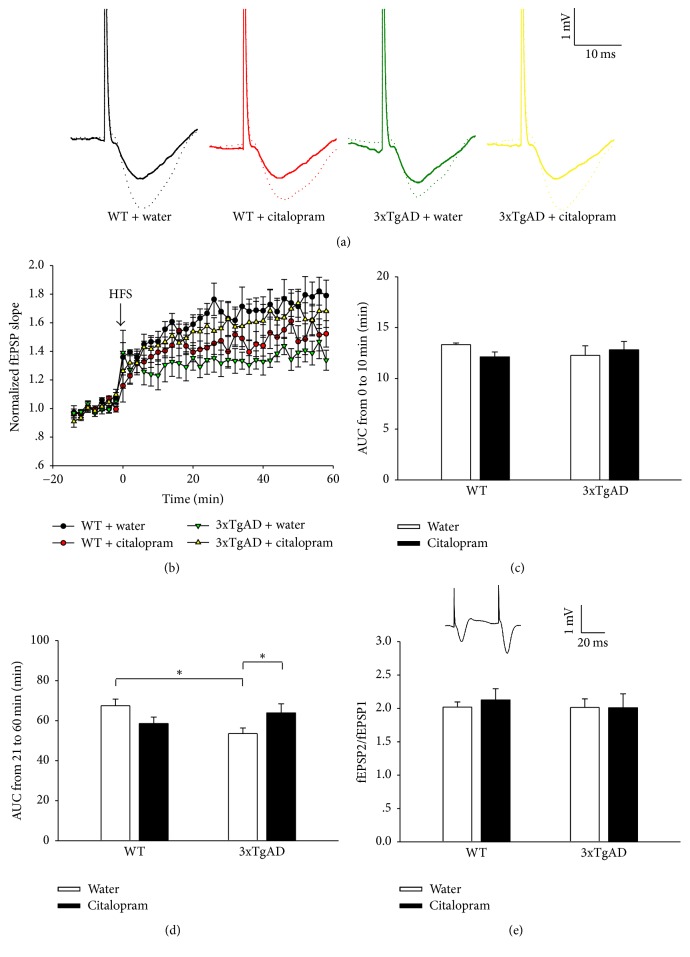
Citalopram treatment rescued hippocampal LTP of 3xTgAD mice. (a) Sample traces of fEPSPs before HFS (solid line) and 60 min after HFS (dotted line). (b) Time course of fEPSPs and LTP induction before and after HFS. (c) Genotype or citalopram treatment did not affect the induction of LTP. (d) Histograms show that the depressive LTP in 3xTgAD mice was reversed by citalopram. (e) Genotype or citalopram treatment did not affect the PPF (fEPSP2/fEPSP1) in the hippocampal CA1 region. Inset, representative paired fEPSPs. Error bars indicate SEM. ^*∗*^*P* < 0.05.

Hippocampal LTP in the CA1 region of mice was recorded to illuminate the possible mechanism underlying the effects of citalopram on learning and memory. The change in fEPSP slope was used to represent the synaptic efficacy in the CA1 region. Sample traces of fEPSPs before HFS and 60 min after HFS are shown in Figure 4(a).  Figure 4(b) illustrates the time-course response. Two-way ANOVA for AUC from 0 to 10 min produced no significant results (*F*_(1,17)_ = 0.0662, *P* = 0.800 for genotype, *F*_(1,17)_ = 0.216, *P* = 0.648 for citalopram, and *F*_(1,17)_ = 1.487, *P* = 0.239 for the interaction between genotype and citalopram; Figure 4(c)), which suggested that genotype and citalopram treatment did not affect the induction of LTP. Two-way ANOVA for AUC from 21 to 60 min showed that genotype or citalopram treatment did not have significant main effect on AUC and a significant interaction between genotype and citalopram existed (*F*_(1,17)_ = 1.512, *P* = 0.236 for genotype, *F*_(1,17)_ = 0.0405, *P* = 0.843 for citalopram, and *F*_(1,17)_ = 7.585, *P* = 0.014 for the interaction between genotype and citalopram). Post hoc analysis indicated that LTP was significantly impaired in 3xTgAD mice (*P* = 0.010, *n* = 5-6; Figure 4(d)), and citalopram reversed the effect (*P* = 0.047, *n* = 5-6).

Before HFS, PPF was examined to clarify whether a presynaptic mechanism was involved in the effects of genotype and citalopram on synaptic plasticity. Two-way ANOVA showed that genotype and citalopram had no significant main effects on the PPF (*F*_(1,17)_ = 0.159, *P* = 0.695 for genotype, *F*_(1,17)_ = 0.0405, *P* = 0.843 for citalopram, and *F*_(1,17)_ = 7.585, *P* = 0.014 for the interaction between genotype and citalopram; Figure 4(e)).

### 3.3. Citalopram Treatment Decreased A*β* Accumulation of 3xTgAD Mice

**Figure 5:**
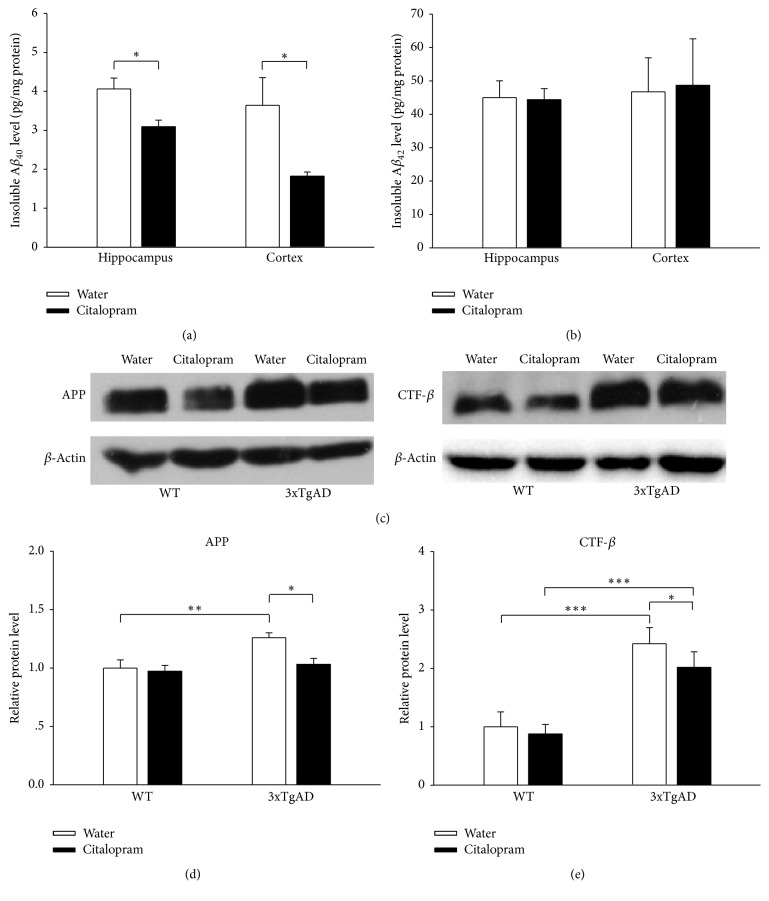
Citalopram treatment decreased A*β* production by inhibiting APP expression in 3xTgAD mice. (a) The insoluble A*β*_40_ concentrations in the hippocampus and cerebral cortex samples from citalopram-treated 3xTgAD mice were significantly lower than the concentrations in water-treated 3xTgAD mice. (b) Citalopram treatment did not have significant effect on the levels of insoluble A*β*_42_ in the hippocampus and cerebral cortex. (c) Representative western blot images of APP and CTF*β* in different groups. (d) Citalopram treatment downregulated APP expression in 3xTgAD mice. (e) Citalopram treatment decreased CTF*β* levels in 3xTgAD mice. Error bars indicate SEM. ^*∗*^*P* < 0.05, ^*∗∗*^*P* < 0.01, and ^*∗∗∗*^*P* < 0.001.

The amount of A*β* immunoreactivity in the hippocampus and cerebral cortex of 3xTgAD mice increased with age [15]. A*β*_40_ and A*β*_42_ levels in samples of hippocampus and cerebral cortex of 3xTgAD mice were measured using an ELISA method to determine whether citalopram treatment suppressed the production of A*β*. Levels of detergent soluble A*β*_40_ and A*β*_42_ were below the limit of detection in hippocampus and cerebral cortex samples in 3xTgAD mice. The insoluble A*β*_40_ concentrations in the hippocampus and cerebral cortex samples from citalopram-treated 3xTgAD mice were significantly lower than the concentrations in water-treated 3xTgAD mice (*P* = 0.018 and *P* = 0.0348, *n* = 5; Figure 5(a)). However, citalopram did not have significant effect on the levels of insoluble A*β*_42_ in the hippocampus and cerebral cortex (*P* = 0.925 and *P* = 0.913, *n* = 5; Figure 5(b)).

### 3.4. Citalopram Treatment Lowered Levels of APP and CTF*β* in Hippocampus of 3xTgAD Mice

A*β* is produced from APP by sequential enzymatic cleavages. To investigate the probable mechanism of how citalopram treatment decreased A*β* accumulation, APP and CTF*β* levels were analyzed by western blotting (Figure 5(c)). Two-way ANOVA showed that genotype, but not citalopram, had significant main effect on the APP levels in hippocampus (*F*_(1,18)_ = 12.456, *P* = 0.003 for genotype, *F*_(1,18)_ = 3.334, *P* = 0.087 for citalopram, and *F*_(1,18)_ = 3.581, *P* = 0.077 for the interaction between genotype and citalopram). Post hoc analysis indicated that the levels of APP in 3xTgAD mice were higher than that in WT mice (*P* = 0.001, *n* = 5; Figure 5(d)), and citalopram treatment significantly decreased the amount of APP in 3xTgAD mice (*P* = 0.018, *n* = 5). Two-way ANOVA of CTF*β* levels showed that genotype and citalopram had significant main effects on the CTF*β* levels in hippocampus (*F*_(1,18)_ = 60.433, *P* < 0.001 for genotype, *F*_(1,18)_ = 4.665, *P* = 0.046 for citalopram, and *F*_(1,18)_ = 1.963, *P* = 0.180 for the interaction between genotype and citalopram). Post hoc analysis revealed that the levels of CTF*β* in 3xTgAD mice were higher than those in WT mice (*P* < 0.001, *n* = 5; Figure 5(e)), and citalopram treatment significantly decreased the levels of CTF*β* in 3xTgAD mice (*P* = 0.023, *n* = 5). After normalizing the levels of APP, citalopram did not change the CTF*β* levels significantly in 3xTgAD mice (*P* = 0.544, *n* = 5). This indicated that the decrease of CTF*β* levels could be due to decreased APP expression.

## 4. Discussion

So far as we know, this study is the first report regarding the beneficial effects of citalopram on the AD-like behaviors of a transgenic AD mouse model. In female 3xTgAD mice, administration of citalopram at approximately 10 mg/kg/day for 2 to 3 months significantly improved spatial memory, hippocampal synaptic plasticity, and A*β* accumulation in the brain.

Citalopram is an effective and well-tolerated treatment for elderly depressed patients with or without dementia [26]. In AD patients, citalopram improved behavioral symptoms, such as agitation and irritability [27, 28]. Also, citalopram can decrease A*β* production in mice and humans [8, 9, 29]. This effect may be mediated by 5-HT4R, 5-HT6R, and 5-HT7R, as the agonists of these receptors could also significantly reduce A*β* production in AD mouse models [29–32]. Similarly, other SSRIs, such as paroxetine and fluoxetine, also decreased A*β* production in AD mouse models [17, 33]. Previous studies were performed on APP/PS1 mice, another model of AD, regarding effect of citalopram [8, 9, 29] and the present study found that the treatment decreased A*β* accumulation in 3xTgAD mouse model for the first time. Interestingly, citalopram treatment lowered insoluble A*β*_40_ levels and did not change insoluble A*β*_42_ levels. However, in APP/PS1 mice, chronic treatment with citalopram reduced insoluble A*β*_40_ and A*β*_42_ concentrations [8]. The 42/40 ratio of A*β* is affected by different *γ*-secretases [34] and mutations in presenilin-1 [35] and other factors. Citalopram could also change the translation of presenilin-1 [8]. Thus, the detailed mechanism why citalopram treatment did not lower insoluble A*β*_42_ levels is complex. Different genotypes may account for the different effects. In 3xTgAD mice, paroxetine also did not decrease A*β*_42_ levels while lowering A*β*_40_ levels [17]. The mechanisms of citalopram-mediated A*β* decrease are complex. In the present study, although citalopram treatment reduced the level of CTF*β* and APP in the hippocampus of 3xTgAD mice, the decreased CTF*β* is not equivalent to decreased A*β* because increased *γ*-secretase enzymatic activity would lead to decreased CTF*β* and increased A*β*. Moreover, after normalizing the levels of APP, citalopram did not show significant change in the CTF*β* levels. Accordingly, it is probable that citalopram primarily affect the accumulation, but not the production, of A*β* in the condition. In addition, citalopram treatment might decrease A*β* accumulation by inhibiting APP expression or increasing *α*-secretase enzymatic activity in 3xTgAD mice. Indeed, it reported that citalopram treatment increased *α*-secretase enzymatic activity and reduced A*β* levels [8, 29]; SSP-002392, a 5-HT4 receptor agonist, decreased APP production and increased *α*-secretase shedding of APP in APP/PS1 mice [32]. Therefore, further researches including measuring several APP catenases such as *α*-secretase and APP metabolic products such as sAPP*β* are still necessary in the future to clarify the mechanism by which citalopram decreases accumulated A*β*.

At the age of 7 months, female 3xTgAD mice showed spatial memory impairment in MWM test, which was in accordance with the previous studies [16, 36]. The impairment was significantly ameliorated by 2-month treatment of citalopram, indicating that the therapy worked for memory improving in 3xTgAD mice. Other SSRIs also showed protective effects on cognition in transgenic mouse models of AD. Paroxetine treatment decreased A*β* levels and improved spatial memory in 3xTgAD mice [17]. Treatment with fluoxetine could also ameliorate spatial memory by suppressing the production of A*β* in APP/PS1 mice [33]. These results suggested that the antidepressants of SSRI class might be potential drugs for memory improvement in AD patients. We noticed that citalopram did not affect performance of 3xTgAD mice in OFT, EPM test, and TST, which suggests that the protective effect of citalopram on spatial memory in 3xTgAD mice was likely due to an effect on cognition rather than an anxiolytic or antidepressive action.

In OFT, 3xTgAD mice showed reduced motor activity. Hypoactivity in OFT was previously reported in this model at 6 or 7 months of age [17, 37]. However, 3xTgAD mice exhibited normal motor ability in danger in MWM test. Reduced spontaneous activity is considered as apathy-like behavior [38]. In mild AD patients, apathy prevalence was 41.6% [39]. However, citalopram did not have effect on apathy-like behavior in our study. In contrast, citalopram treatment increased locomotor activity in WT mice. Acute administration of citalopram at the dose of 10 mg/kg could also lead to hyperactivity of WT mice in OFT [40]. This effect might result from activation of 5-HT_1B_ and 5-HT_2A_, as agonists of which could also increase locomotor activity in WT mice [40]. In our study, 3xTgAD mice spent more time in inner square in OFT and showed a lower level of anxiety at this age. 3xTg AD mice between 12 and 14 months of age also spent more time in inner square (*P* = 0.08) [41]. Some other studies focusing on OFT in 3xTgAD mice did not measure the index [17, 37, 42–44]. In EPM test, 3xTgAD mice also showed reduced motor activity and a lower level of anxiety, which was consistent with the findings in OFT. Citalopram treatment did not change the abnormalities in OFT. In mild AD patients, depression had a prevalence of 47.9% [39]. 18-month-old male 3xTg-AD mice also showed depressive-like behavior [44]. But at the age of 8 months, female 3xTg-AD mice did not present the behavior in our study. Young age might account for the difference.

Hippocampal LTP is considered as a cellular model of synaptic plasticity and learning and memory formation. At the age of 6 months, 3xTgAD mice exhibit hippocampal LTP impairment accompanied by deficits in spatial learning and memory [15]. Our results confirmed the depression of LTP in 3xTgAD mice and chronic citalopram treatment reversed it. There are at least two mechanisms explaining the effect of citalopram. First, A*β* impairs hippocampal LTP [45, 46] and citalopram decreases its accumulation. Second, serotonin concentrations are reduced in hippocampal cortex and hippocampus [12], and depletion of 5-HT in vivo results in impairment of LTP [47, 48]. Citalopram can increase hippocampal 5-HT levels in mice [14] and thus may restore LTP. However, excess 5-HT depresses LTP. In rat brain slices, bath-applied 5-HT blocked hippocampal LTP [49, 50]. SSRIs also suppress hippocampal LTP of rats [51–53]. In our study, the LTP of WT mice treated with citalopram tended to be depressive (*P* = 0.095). High hippocampal 5-HT levels beyond normal range may account for the effect. Accordingly, spatial learning ability (*P* = 0.029, on the fifth training day) and memory (*P* = 0.066) of WT mice receiving citalopram also tended to deteriorate in MWM test. Similar effect was found in paroxetine treated WT mice [17].

Although citalopram improved cognition in 3xTgAD mice, the effect on AD patients is not determined. The CitAD (The Citalopram for Agitation in Alzheimer Disease) study, a randomized, placebo-controlled, double-blind clinical trial, showed that citalopram had a small negative effect on cognitive functioning [27]. However, the effect does not achieve a minimum clinically significant change [54] and its clinical significance is uncertain [27]. In addition, the subjects in the CitAD study had agitation of moderate severity and the sample cannot fully represent AD patients. The inconsistent effects may also result from different stages of disease. Seven-month-old 3xTgAD mice are at very early stage of AD [15]. In contrast, The CitAD study included AD patients having moderate dementia. In our study, mice receiving higher dose of citalopram and different dose may also account for the contradictory effects.

## 5. Conclusions

In this study, we tested the effects of citalopram on behavioral performance and synaptic plasticity in female 3xTgAD mice. Chronic administration of citalopram significantly improved spatial memory and synaptic plasticity and decreased insoluble A*β* levels in female 3xTgAD mice. Taken all together, citalopram treatment may be a promising strategy for AD and further clinical trials should be conducted to verify the effect of citalopram on cognition in patients with AD or mild cognitive impairment.
